# Repair Bond Strength and Leakage of Non-Aged and Aged Bulk-fill Composite

**DOI:** 10.3290/j.ohpd.a45082

**Published:** 2020-09-04

**Authors:** Cintia Aquino, Caroline Mathias, Suelem Chasse Barreto, Andrea Nóbrega Cavalcanti, Giselle Maria Marchi, Paula Mathias

**Affiliations:** a MSc Student, Department of Biochemistry and Biophysics, Institute of Health Sciences, Federal University of Bahia, Salvador, Bahia, Brazil. Performed the experiments in partial fulfillment of requirements for a MSc degree, wrote the manuscript.; b Professor, Department of Restorative Dentistry of Faculty of Technology and Sciences (UniFTC), Salvador, BA, Brazil. Idea, experimental design, proofread the manuscript, performed μTBS, and thermocycling tests.; c Dentist, Brazilian Army, Rio de Janeiro, Brazil. Proofread the manuscript, performed leakage analysis and μTBS test.; d Professor, Department of Clinical Dentistry, School of Dentistry, Federal University of Bahia, Salvador, Bahia, Brazil. Interpreted data, proofread the manuscript, performed statistical evaluation, contributed substantially to discussion.; e Professor, Department of Restorative Dentistry, Piracicaba Dental School, State University of Campinas, Piracicaba, São Paulo, Brazil. Proofread the manuscript, contributed substantially to discussion.; f Professor, Department of Clinical Dentistry, School of Dentistry, Federal University of Bahia, Salvador, Bahia, Brazil. Idea, experimental design, hypothesis, contributed substantially to discussion, critical revision of the manuscript.

**Keywords:** aging, composite resins, repair, sandblasting, silane

## Abstract

**Purpose:**

This study evaluated repair protocols of a non-aged and aged bulk-fill composite in terms of bond strength and leakage.

**Materials and Methods:**

Ninety-six bulk-fill resin specimens were constructed; half were submitted to thermocycling. Specimens were divided into six groups (n = 16) according to the repair treatments: CG: no repair (control group); Ad: adhesive; DbAd: abrasion with diamond bur + adhesive; SbAd: sandblasting + adhesive; DbSiAd: abrasion with diamond bur + silane + adhesive; and SbSiAd: sandblasting + silane + adhesive. Resin blocks were bonded to the treated surfaces to simulate repair, and the specimens were submitted to microtensile bond strength testing. The failure area was evaluated under a stereomicroscope (40X magnification), and leakage after specimen immersion in silver nitrate solution for 24 h was evaluated under a microscope (200X magnification). Three-way ANOVA (surface treatment, chemical agent, aging) and Tukey’s test were performed.

**Results:**

Ad and DbAd groups showed the lowest bond strengths, while Ad was the only group negatively influenced by aging. The other groups were statistically similar to the CG in both conditions. All groups exhibited leakage, but groups without silane presented a greater percentage of leakage, mainly when diamond burs were used. Thermocycling did not influence leakage, nor did surface treatment in groups with silane.

**Conclusion:**

For composite repair, the use of silane is recommended, mainly when diamond burs are used as a mechanical surface treatment.

The mechanical, aesthetic, and functional properties of composite resins have made these materials a good option for anterior and posterior direct restorations.^4,32^ However, although widely used, composite resin restorations still fail, necessitating a decision whether to replace or repair the restoration.^15^ Some clinical aspects should be considered in the decision to completely replace or repair the restoration. When there is secondary caries or total debonding of the restoration, complete replacement should be advocated. On the other hand, when there are small fractures with sound margins or localised stains, the repair of the composite resin is indicated.^15,33^ Repairing a restoration is a minimally invasive procedure in which the defective portion of the composite is removed and a new composite material is added to complement the restoration.^33^

Considering the clinical consequences, some authors have suggested that it is better to repair defective restorations than to totally replace them.^10,15^ The advantages of repairing a composite restoration include: decreased removal of remaining tooth structure, reduced incidence of pulpal injury, and lower cost.^1,8^ The repair might improve the clinical longevity of restoration, and should be indicated for localised defects and clinically unsatisfactory portions of restorations, including superficial marginal discolouration, colour correction, restoration fracture, or tooth fracture.^15,33^

The major challenge for composite resin repair is the difficulty of bonding between aged and fresh resin, since resin undergoes changes after its insertion into the mouth, such as water absorption, chemical degradation, and loss of inorganic particles.^11^ Water absorption occurs due to chemical degradation of the ester groups, mainly through salivary esterase. Water soption may also occur through the hydrophilic groups (OH) in some cross-linking resin monomers.^7^ When exposed to a very moist environment such as the oral cavity, monomers degraded due to weakening the bond between the inorganic particles and resin matrix.^16^ This process also leads to softening of the resin matrix by swelling of the polymer network, decreasing the mechanical properties and negatively influencing the adhesion of the material.^16^

The surface of a restoration consists of an organic matrix that has already matured, lacks unreacted double bonds (C=C), and has thus become less reactive; additionally, the filler particles are devoid of silane and are unable to chemically bond to a new composite.^1^ Several studies have investigated different surface treatments of conventional resins to assess the bond strength between the aged composite resin and the newly applied resin.^23,26,27,30^ These surface treatments remove the superficial layer of an existing composite that has become altered by exposure to the oral environment, increasing the surface energy and the surface area of the resin.^10^ However, studies using several types of resin composite have shown controversial and inconclusive results on which surface treatement is best.^27^

Mechanical surface treatment involves roughening the resin surface using diamond burs or air abrasion with aluminum oxide particles to remove the aged surface resin.^23^ Commonly, phosphoric acid (37%) is used to clean the surface of the composite resin to be repaired,^11^ while the use of silane promotes chemical bonding to the filler particles of the resin, increasing the flowability of the adhesive on irregular surfaces.^31^ The adhesive is responsible for chemical bonding to the organic matrix of the resin composite serving as the intermediate bonding agent that increases the wettability of the fresh composite resin.^23^

Bulk-fill (monoincremental) resins have lower polymerisation shrinkage than conventional resins, and they have the advantage of polymerising at depths of up to 5 mm.^30^ These polymerisation depths can be attributed to the greater translucency of such materials, allowing photoactivation of thicker resin increments.^17^ Other modifications have been made in the composition of bulk-fill resins, such as the use of specific monomers that modulate the polymerisation reaction and thus relieve stress, the use of more reactive photoinitiators, in addition to the use of different inorganic particles, such as prepolymer particles and segments of fiberglass.^17^

When considering the increasing and promising use of bulk-fill resin composites, concerns regarding the durability and factors related to the survival and clinical success of these composites remain, since they are commonly used in extensive, deep restorations (4-5 mm deep in posterior teeth), where repair would bring numerous benefits, when well indicated. Due to the lack of information in the literature about adequate repair protocols for bulk-fill resins, it is necessary to assess and identify the best repair protocol for these resins, since they have different compositions. The aims of this study were to evaluate a. evaluate different repair protocols on the bond strength of a bulk-fill resin composite before and after thermocycling, b. the fracture mode of repaired resin composites, and c. the leakage of the adhesive interface of repaired resins composites. Thus, the null hypotheses of this study were: (1) The type of repair protocol does not influence the bond strength of a repaired bulk-fill composite; (2) the aging process influences the bond strength of bulk-fill composite regardless of the repair protocol used.

## Materials and Methods

### Experimental Design

**Fig 1:**
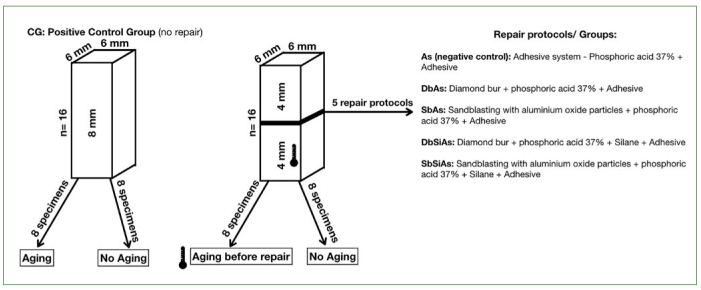
Experimental design showing the blocks made for the positive control group and blocks made for repaired groups. Thermometer: only 8 of the blocks initially made from the repaired groups underwent thermocycling, thus simulating an aged resin that would later receive a repair.

The experimental design is shown in Fig 1. Bulk-fill resin blocks (Filtek Bulk Fill, shade B1, 3M Oral Care; St Paul, MN, USA) were made using two metallic matrices. One matrix measuring 6 x 6 x 8 mm was used to make single blocks for the positive control group (CG, n = 16) which did not receive repair. The CG group was fabricated by using two bulk-fill increments (4 mm each) using a metal spatula (Goldstein XTS flex, Hu-Friedy; Chicago, IL, USA) in order to obtain the 8-mm high specimens. After insertion and adaptation of each increment, the resin was covered with a polyester strip and a glass slide under 500-g weight for 60 s. The samples were light cured using a light emitting diode (LED) (Valo, Ultradent; South Jordan, UT, USA) at an irradiance of 1000 mW/cm^2^ for 40 s. For the other groups, 80 bulk-fill single increment (4 mm) was made using a metallic matrix measuring 6 x 6 x 4mm, processed in the same manner as described above. Afterwards, these blocks were divided into 5 groups (n = 16).

Half of the specimens of all groups were submitted to thermocycling (5±1°C, 37±1°C and 55±1°C), dwell time 30 s per bath, with an interval of 15 s using a 3-chamber thermal device (MSCT-3e, Elquip; São Carlos, SP, Brazil). Thus, the samples were divided into two subgroups: Aged (A) and non-aged (Na).

Subsequently, the specimens (except the positive control group) were submitted to 5 different repair protocols:

- Ad (negative control, no mechanical or chemical surface treatment was applied): etching with 37% phosphoric acid, then adhesive application.
- DbAd: diamond bur followed by 37% phosphoric acid etching, then adhesive application.
- SbAd: sandblasting with aluminum oxide particles followed by 37% phosphoric acid etching, then adhesive application.
- DbSiAd: diamond bur followed by 37% phosphoric acid etching, then silane and adhesive application.
- SbSiAd: sandblasting with aluminum oxide particles followed by 37% phosphoric acid etching, then silane and adhesive application.

For the application of adhesive in the negative control and experimental groups, the surface of each specimen was etched using 37% phosphoric acid (Condac 37, FGM; Joinvile, SC, Brazil) for 30 s, then the specimenswere washed with distilled water for 15 s and air dried. One drop of Adper Single Bond 2 adhesive (3M Oral Care) was applied to the entire surface, the adhesive solvent was evaporated for 10 s, after which light cured was performed for 10 s.

In groups DbAd and DbSiAd, the surface was mechanically treated using a cylindrical diamond bur #4138 (KG Sorensen; Cotia, SP, Brazil) that was kept in contact with the surface for 5 s at high speed under air/water cooling. The diamond bur was replaced after every five specimens.

For groups SbAd and SbSiAd, the specimens were sandblasted with 50-µm Al_2_O_3_ particles (Bio-Art, São Carlos, SP, Brazil) at a distance of 10 mm at a 90-degree angle and 4.1 bar pressure for 10 s.

For groups DbSiAd and SbSiAd that received chemical surface treatment, the silane (Prosil, FGM) was applied on the surface of the specimens for 1 min, which were then air dried for 10 s to evaporate the solvent.

To perform the repair procedure, 4-mm blocks were inserted into the metallic matrix (6 x 6 x 8mm) after the respective surface treatments, and then the specimens were repaired using new bulk-fill resin blocks (Filtek Bulk Fill, shade A3, 3M Oral Care), which were fabricated as described above. The specimens were stored in distilled water at 37°C for 24 h.

### Microtensile Bond Strength Test (μTBS)

Blocks were longitudinally sectioned in both the “x” and “y” directions across the bonded interface using a diamond blade (Buehler; Lake Bluff, IL, USA) in a high-speed precision saw (Isomet 1000, Buehler; Lake Bluff, IL, USA) under constant irrigation. This process resulted in 25 sticks with a cross-sectional area of approximately 1.0 mm^2^. Sticks from the external area were excluded.

Sticks were individually fixed using cyanoacrylate glue (Super Bond gel, Loctite, Henkel; São Paulo, SP, Brazil) to a custom-made apparatus in a Universal Testing Machine (EZ Test L, Shimadzu; São Paulo, SP, Brazil) using an approximately 500-kgf load cell at a crosshead speed of 1.0 mm/min until failure. The cross-sectional area at the site of fracture was measured using a digital caliper (Mitutoyo; Tokyo, Japan) and bond strengths were reported in MPa. Thus, the load at failure was calculated considering the dimensions of the sample at the adhesive interface, according to the formula: F (MPa) = load at failure (N)/area (mm^2^)

### Failure Mode

After μTBS testing, one specimen from each group was evaluated using a stereoscopic magnifying glass (Opton, NTB-3A; Cotia, SP, Brazil). Images were obtained at 40X magnification to determine failure mode, which was classified as adhesive, mixed, or cohesive.

### Leakage Analysis

Two sticks from each group (except the positive control group) were immersed in a 50% ammoniac silver nitrate solution in total darkness for 24 h, washed in distilled water, and immersed in a photo-developing solution for 8 h under fluorescent light to reduce ions into metallic silver grains along the interface.^3^ The sticks were then embedded in polystyrene resin and polished with 600-, 1200-, and 2000-grit SiC sandpaper and 3-, 1-, and 0.25-μm diamond paste (Arotec; São Paulo, SP, Brazil). Specimens were ultrasonically cleaned (Ultrasound Ultrason 1440 D, Odontobrás; Rio Preto, SP, Brazil) in distilled water for 10 min between each application of sandpaper and felt disks, as well as at the end of the polishing treatment in order to remove the polishing debris.^3^

The specimens were inspected using an optical microscope (Carl Zeiss, Scope A1/AXIO; Jena, Germany) at 200X magnification allowing visualisation of the entire repaired area. Images were recorded to quantitatively assess the infiltrated area using ImageJ software (ImageJ, National Institute of Health; Bethesda, MD, USA). The extent of the area infiltrated by ammoniacal silver nitrate solution was obtained in millimeters, which were then converted to a percentage considering 100% as the total area of the bonded interface.

### Statistical Analysis

The μTBS and leakage data were confirmed for normality using the Kolmogorov-Smirnov test. Then, a three-way ANOVA followed by Tukey’s post-hoc HSD test for multiple comparisons was performed to analyse the effects of surface treatment (diamond bur or sandblasting), chemical agent (presence of silane or not), and aging (thermocycling or not). One-way ANOVA followed by Dunnett’s test was used to individually compare the μTBS and leakage obtained in the positive and negative control groups against the experimental repair protocols. Statistical signiﬁcance was set at α = 0.05. Statistical analysis was carried out using SAS 9.1 software (SAS Institute; Cary, NC, USA).

## Results

### Microtensile Bond Strength Test

The mean μTBS of all groups are summarised in Table 1.

**Table 1:** Repair bond strength means (SD) in MPa by experimental group

Surface treatment	Aging	Chemical agent	
		Without silane	With silane
Diamond bur + adhesive	Non-aged	26.48 (4.29)^Bb^	32.92 (5.80)^Aa^
	Aged	26.28 (2.29)^Bb^	32.70 (4.15)^Aa^
Sandblasting with Al_2_O_3_ particles + adhesive	Non-aged	32.78 (5.99)^Aa^	33.84 (5.30)^Aa^
	Aged	32.20 (4.37)^Aa^	31.88 (3.89)^Aa^

Same superscript uppercase letters within columns indicate no signiﬁcant differences (three-way ANOVA followed by Tukey’s post-hoc multiple comparisons) between the surface treatments. Same superscript lowercase letters within rows indicate no signiﬁcant differences between the chemical agents. No statistically signiﬁcant difference was found between the aged and the non-aged specimens.

Three-way ANOVA did not demonstrate a significant interaction between the three main factors (surface treatment x chemical agent x aging) (p = 0.77). However, a statistically significant interaction was noted between surface treatment and chemical agent (p = 0.01; Tukey’s test, Table 1). The main factor ‘aging’ was analysed separately, and no significant difference was noted (p = 0.57).

When considering the factor ‘chemical agent’, there was a statistically significant difference for the diamond bur treatment. In this case, DbAd showed lower µTBS (26.48 ± 4.29 MPa) than did DbSiAd (32.92 ± 5.80 MPa). Furthermore, there was a difference for the surface treatment factor in the absence of silane, where higher bond strengths were found for the sandblasted groups (SbAd, 32.78 ± 5.99 MPa).

**Fig 2:**
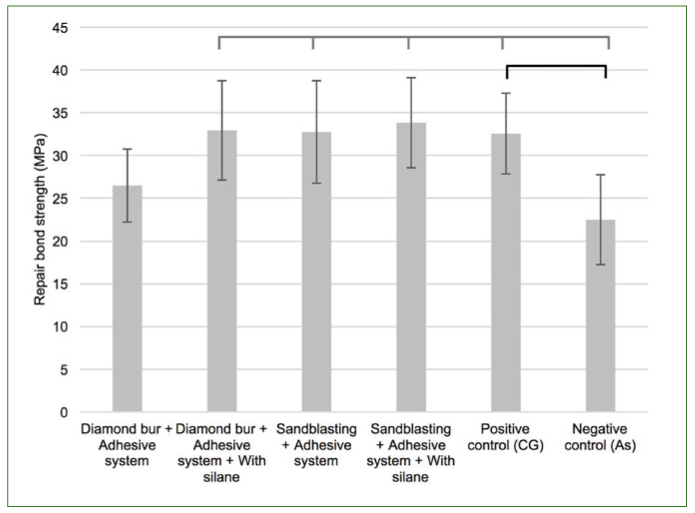
Microtensile bond strength of non-aged controls compared using one-way ANOVA followed by Dunnett’s test. Horizontal lines above bars represent significant differences between paired groups (p < 0.05). Gray bracket-bar represents comparisons with the negative control, As group. Black bracket-bar represents comparisons with the positive control, CG.

**Fig 3:**
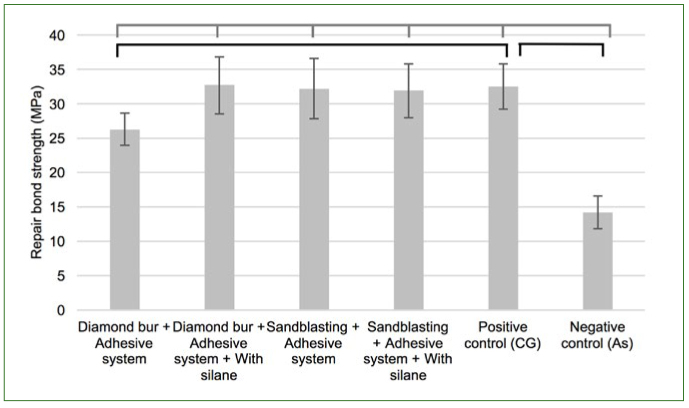
Microtensile bond strength of aged controls compared with one-way ANOVA followed by Dunnett test. Horizontal lines above bars represent significant differences between paired groups (p < 0.05). Gray bracket-bar represents comparisons with the negative control, Ad group. Black bracket-bars represents comparisons with the positive control, CG.

Dunnett’s test showed statistically significant differences between control groups (negative and positive) and experimental groups (p < 0.001) under non-aged and aged conditions (Figs 2 and 3, respectively). The negative control, Ad, had the lowest bond strength among the non-aged groups, which was statistically significantly different from all conditions, except DbAd. On the other hand, the non-aged positive control, CG, only presented a statistically significant difference compared to the negative control, being similar to the other conditions (Fig 2).

The aged negative control, Ad, presented the lowest bond strength among the aged groups. However, the aged positive control, CG, was only statistically different from the aged Ad group and DbAd group (Fig 3).

### Failure Mode

**Fig 4:**
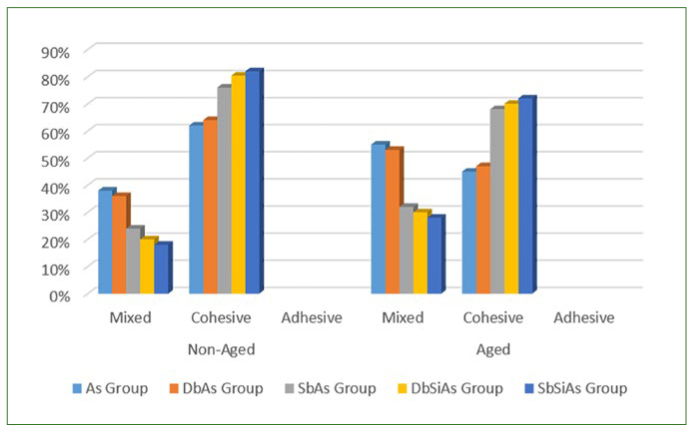
Failure mode distribution according to groups.

**Fig 5:**
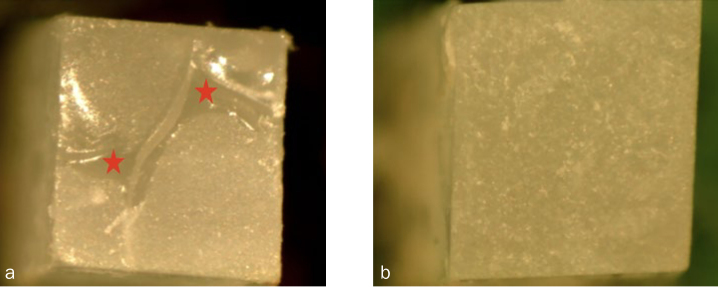
Representative images of failure modes. a) Mixed failure: adhesive (shiny portions indicated by red stars) and composite resin (dull portions) are present. b) Cohesive failure: no adhesive is apparent, just the composite resin (dull uniform fracture surface) is present.

Failure mode frequencies were analysed (Fig 4). The most common failure mode was cohesive (Fig 5b). However, mixed failure (Fig 5a) was predominant for groups Ad and DbAd groups. For all aged groups tested, the rate of cohesive failures decreased and the mixed failures increased, especially for groups Ad and DbAd.

### Leakage

**Fig 6:**
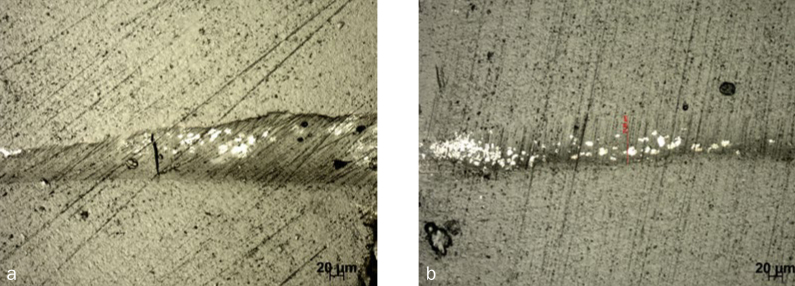
Representative image of leakage. a) Non-aged SbAd group showing few areas infiltrated by silver nitrate solution (4.67 ± 2.87%) compared to b) non-aged Ad group showing greater infiltration by silver nitrate solution (12.85 ± 1.70).

All of the groups tested showed silver nitrate infiltration. A significant interaction among the three main factors could not be detected by the three-way ANOVA (p = 0.44). But the interaction between surface treatment and chemical agent was statistically significant (p < 0.001) and the differences between the levels were compared with Tukey’s test (Table 2). The main factor ‘aging’ was analysed separately, and no statistically significant difference was noted (p = 0.14). Representative images of leakage are depicted in Fig 6.

**Table 2:** Interaction between mechanical treatment factor and silane (aging data are grouped) for leakage (%)

Surface treatment	No silane	Silane
Diamond bur + adhesive	12.01 (2.37)^Aa^	2.37 (0.85)^Ab^
Sandblasting with Al_2_O_3_ particles + adhesive	4.54 (2.49)^Ba^	2.84 (1.06)^Aa^

Means followed by different superscript letters indicate statistically significant differences (3-way ANOVA, Tukey’s test). Superscript uppercase letters compare surface treatment factors. Superscript lowercase letters compare the silane factor (with/without).

When surface treatment with a diamond bur was considered, the use of silane statistically significantly decreased the leakage values (2.37 ± 0.85%). On the other hand, the use of silane did not statistically significantly change the percentage of leakage between sandblasted groups. In the absence of silane, groups treated with diamond burs presented a statistically significantly higher percentage of leakage (12.01± 2.37%) compared to group SbAd (4.54 ± 2.49%, Fig 6a). However, when silane was used, there was no difference in leakage values between the mechanical surface treatments.

**Fig 7:**
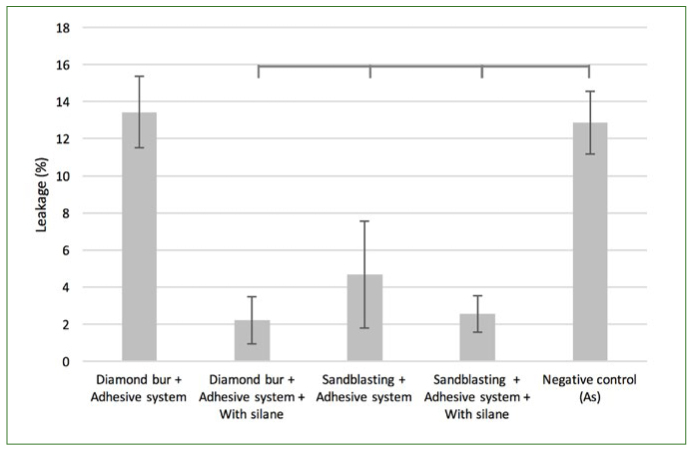
Percentage of leakage in non-aged controls compared using one-way ANOVA followed by Dunnett’s test. Horizontal lines above bars represent significant differences between paired groups (p < 0.05).

**Fig 8:**
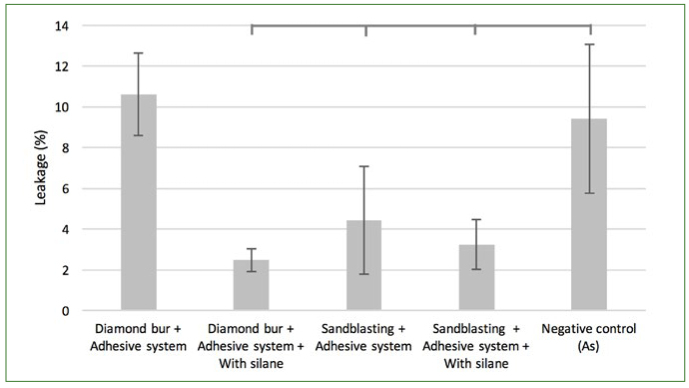
Percentage of leakage in aged controls compared using one-way ANOVA followed by Dunnett’s test. Horizontal lines above bars represent significant differences between paired groups (p < 0.05).

Dunnett’s test showed a significant difference between the non-aged Ad and the other experimental groups (p < 0.001), with the exception of DbAd (Fig 7). The same difference was noted within aged groups (Fig 8).

## Discussion

The first null hypothesis of this study was rejected, since there were differences among the surface treatments studied, considering that groups Ad and DbAd were different from the other groups. The second null hypothesis was also rejected, since the aging process did not influence the bond strength provided by experimental repair procedures.

Group Ad presented the lowest repair bond strength for the bulk-fill resin in both non-aged and aged conditions. This could be due to a remaining resin-aged layer, which would have no unreacted double bonds, resulting in decreased bonding to the new resin. Surfaces submitted to the aging process presented even worse results for this group, due to a more pronounced monomer chemical degradation and absorption of water, in addition to the loss of inorganic particles.^16^ Also, as adhesives are more hydrophilic, they have a greater degradation potential.^13^ This finding corroborates the study by Melo et al,^25^ which concluded that the adhesive (37% phosphoric acid plus adhesive) should not be used alone as a surface treatment in repair protocols of an aged microhybrid resin. The use of phosphoric acid is not capable of creating microretentions on a composite resin surface;^25^ it is used with mechanical treatment only to remove debris from the surface to be repaired, exposing the irregularities of the surface and increasing the available total area for adhesion.^11^

With regard to the mechanical surface treatment, the use of a diamond bur resulted in lower bond strengths than did the group in which sandblasting was used as a surface treatment. Rodrigues Jr et al^27^ examined bur-treated surfaces of microhybrid and nanoparticulate resins with SEM and found a very irregular and non-retentive surface when a medium-grain diamond bur was used. Those authors^27^ found a more uniform topography on surfaces treated with sandblasting using Al_2_O_3_ particles, which resulted in greater bond strengths, suggesting more effective mechanical retention. However, other studies did not find statistically significant differences between these two types of mechanical treatment.^25,34^ Another study found a decrease in bond strength when surfaces were sandblasted with Al_2_O_3_ particles, attributing this to a reduction of the amount of available resin with excessive exposure of filler particles.^2^ Therefore, finding a balance between surface roughness and the remaining filler particles seems to be a determining factor for optimising repair bond strength.^23,29^

In the present study, diamond bur followed by silane application yielded bond strengths similar to those of groups SbAd and SbSiAd. Therefore, diamond burs can be used to remove the superficial layer of resin if followed by silane application in situations lacking an intraoral sandblaster to provide adequate surface roughness for repair. This process could contribute to increased bond strength of repair for bulk-fill resins. Moreover, intraoral sandblasting with Al_2_O_3_ particles requires the use of absolute isolation with rubber-dam to protect soft tissues and excellent suction to prevent fine abrasive particles from contaminating the environment, which may be harmful to the patient and operator.^23^

Silane has two main functional groups: silanol, which links to silica particles in the resin, and an organofunctional group that links to methacrylates in the adhesive.^23^ Studies on an intermediate layer of silane have obtained conflicting results.^25,30^ Due to its viscosity, greater capacity of surface wetting, and the ability to bond to inorganic components of composites, silane could increase the bond strength of resins.^30^ However, silane has hydrophilic groups which can create a great amount of hydrogen bonds; if applied liberally, it may increase water sorption and hydrolytic degradation over time.^16,24,31^ This degradation may occurs via hydrolysis of the silane chiefly under mildly acidic pHs, resulting in debonding or releasing of the filler particles in composite resins or adhesive.^14^ On the other hand, the bond strength results regarding the use of silane could be explained by the greater wetting and penetration of silane into surface irregularities, improving the repair mechanical properties when silane was applied after using diamond burs on the surface to be repaired. Nevertheless, when the surface was sandblasted with Al_2_O_3_ particles, silane did not improve the bond strength, probably because the sandblasting process depends on the microstructure of composite resin. Thereby, in a nanoparticulate resin such as the bulk-fill resin tested, sandblasting could disrupt filler particles, which reduces their interaction with silane.^27^

Another function of silane is to resilanise inorganic particles on the old surface, improving the bond between the particles in the polymerised composite and the organic matrix of the new composite.^31^ This effect may be dependent on the type of composite repaired, since chemical coupling between silane and composite is influenced by the amount of available silica on the surface.^33^ The bulk-fill resin in the current study is composed of diurethane dimethacrylate (UDMA), aromatic dimethacrylate urethane (AUDMA), dodecane dimethacrylate (DDDMA), and contains about 1-10% by weight silane-treated silica, which may have improved the repair bond strength of group DbSiAd.

It has been estimated that 10,000 thermocycles represent about one year of clinical service.^18^ The literature often mentions decreased bond strength of composite resin after aging, but there are only a few reports on the performance of repaired bulk-fill resins.^6,9,22,30^ Although the process of water absorption is multifactorial, it is largely due to the hydrophilic nature of the monomers composing the polymer.^16^ The lack of decrease in bond strength after aging could be attributed to the composition of Filtek Bulk Fill composite, which contains an additional fragmentation monomer as well as aromatic dimethacrylate urethane, which is a higher molecular-weight monomer than traditional dimethacrylates, and which presents lower water sorption due to the presence of the urethane groups.^21^

Most of the fractures found in this study were cohesive, mainly in the non-aged groups that showed the highest bond strengths (SbAd, DbSiAd and SbSiAd groups). This result may suggest that the bond strength of the repair exceeded the cohesive strength of the resin, and the repair should not be considered as the weakest part of the composite.^34^ There was an increase in mixed fractures after aging, especially in the Ad and DbAd groups. These findings are in accordance with the literature, that higher bond strength leads to a greater number of cohesive fractures and a smaller number of mixed fractures in composites.^5^

Leakage is an important predictor of the interfacial sealing ability by an adhesive material.^28^ The infiltration of oral fluids and bacterial products through the interface can compromise the repair stability in composite resins and can increase staining in this interface. Although no surface treatment has been able to prevent leakage, groups SbAd, DbSiAd, and SbSiAd showed lower leakage than did groups Ad and DbAd, regardless of aging. This result highlights the risk of using only the adhesive or the diamond bur in composite resin repair without silane. These findings agree with another study which also found no difference in leakage levels between aged and non-aged groups of bulk-fill resins that were not repaired.^20^

A repair bond strength between 15 and 25 MPa is considered clinically acceptable, because these are bond strengths given between dentin and resin.^1^ With the exception of group Ad, the other experimental groups had bond strengths above 25 MPa. However, the investigation of bond strength and leakage in the same study allows a more accurate evaluation of the adhesive behavior and the permeability of the bonding surface area of the repair. Thus, the benefits of combined mechanical and chemical surface treatment are confirmed, where the use of only an adhesive should be avoided when repairing bulk-fill resins. Moreover, other surface pretreatments successfully tested on hybrid resin materials, eg, silicatisation through Co-Jet, which presented good repair bond strength^19^ should be considered as possible alternatives for repairing composite resins. Nevertheless, randomised long-term clinical trials should performed in the future to confirm these results, especially in oral conditions where bulk-fill resins may be subject to greater degradation.

## Conclusion

The use of an adhesive without any mechanical treatment is not recommended for bulk-fill resin repair. Also, the use of silane should be recommended to improve bond strength and decrease leakage when a diamond bur is used as the mechanical surface treatment. Aging affected bond strength when the repair was done using only an adhesive. Treatment with aluminum oxide blasting made subsequent use of silane unnecessary. In this study, leakage occurred at all repair interfaces.
